# Transcriptome Analysis Suggests PKD3 Regulates Proliferative Glucose Metabolism, Calcium Homeostasis and Microtubule Dynamics After MEF Spontaneous Immortalization

**DOI:** 10.3390/ijms26020596

**Published:** 2025-01-12

**Authors:** Jocshan Loaiza-Moss, Ursula Braun, Michael Leitges

**Affiliations:** Division of Biomedical Sciences, Faculty of Medicine, Memorial University of Newfoundland, 300 Prince Philip Drive, St. Johns, NL A1B 3V6, Canada; jmloaizamoss@mun.ca (J.L.-M.); ubraun@mun.ca (U.B.)

**Keywords:** protein kinase D3, transcriptome analysis, RNA-seq, spontaneous immortalization

## Abstract

Cell immortalization corresponds to a biologically relevant clinical feature that allows cells to acquire a high proliferative potential during carcinogenesis. In multiple cancer types, Protein Kinase D3 (PKD3) has often been reported as a dysregulated oncogenic kinase that promotes cell proliferation. Using mouse embryonic fibroblasts (MEFs), in a spontaneous immortalization model, PKD3 has been demonstrated as a critical regulator of cell proliferation after immortalization. However, the mechanisms by which PKD3 regulates proliferation in immortalized MEFs require further elucidation. Using a previously validated *Prkd3*-deficient MEF model, we performed a poly-A transcriptomic analysis to identify putative *Prkd3*-regulated biological processes and downstream targets in MEFs after spontaneous immortalization. To this end, differentially expressed genes (DEGs) were identified and further analyzed by gene ontology (GO) enrichment and protein–protein interaction (PPI) network analyses to identify potential hub genes. Our results suggest that *Prkd3* modulates proliferation through the regulation of gene expression associated with glucose metabolism (*Tnf*, *Ucp2*, *Pgam2*, *Angptl4*), calcium homeostasis and transport (*Calcr* and *P2rx7*) and microtubule dynamics (*Stmn2* and *Map10*). These candidate processes and associated genes represent potential mechanisms involved in *Prkd3*-induced proliferation in spontaneously immortalized cells as well as clinical targets in several cancer types.

## 1. Introduction

During spontaneous immortalization, cells acquire the potential to proliferate indefinitely, which represents a clinically significant phenotype common in most human cancers [1,2]. Several of the molecular changes associated with spontaneous immortalization have been implicated as early genetic events in human carcinogenesis [3,4,5,6]. Several human cancers and mutational landscapes relevant to cancer development have been studied using in vitro immortalized cell models, primarily mouse embryonic fibroblasts (MEFs) [7,8,9,10]. Over the years, a number of reports have attempted to explain the general mechanism underlying immortalization of murine fibroblasts, where this process has been associated with genomic instability, mutations and expression changes, including those in the p16*^Ink4a^* and p19*^Arf^* pathways [11,12]. Recently, a transcriptomic study from our group predicted that spontaneous immortalization in MEFs is associated with increased cell proliferation and decreased cell adhesion, which are related to the altered expression of genes such as MAP kinases and E-cadherin [13].

Protein kinase Ds (PKDs) are a family of serine/threonine kinases composed of three isoforms in humans: PKD1 (*PRKD1*, formerly known as PKCμ), PKD2 (*PRKD2*) and PKD3 (*PRKD3*, formerly known as PKCν) [14,15,16]. In general, PKDs have a conserved structure consisting of an N-terminal regulatory domain containing a ubiquitin-like domain that allows for homo- and hetero-dimerization [17], two cysteine-rich zinc finger domains that mediate the localization of PKDs to specific cellular membranes [18], a pleckstrin homology domain involved in the regulation of protein activation and autophosphorylation [19] and a C-terminal kinase effector domain. In general, PKDs act downstream of protein kinase Cs (PKCs) and diacylglycerol (DAG) signaling, which are sensitive to intracellular stimuli such as hormones, growth factors and activators of G-proteins [20,21,22]. As a family, PKDs have been shown to be of great clinical relevance in cancer development and progression due to their involvement in the regulation of cell proliferation, survival, EMT, migration, invasion, angiogenesis regulation and immune response modulation (as reviewed in [23,24]). More specifically, the aberrant expression of *PRKD3* has been identified in cancer cells of various tumor types, including colorectal cancer (CRC), gastric cancer (GC), liver cancer (LC), prostate cancer (PC) and breast cancer (BC) [25,26,27,28,29]. High expression of this protein has been correlated with a poor prognosis, such as in hepatocellular carcinoma (HCC) and progression to invasive PC [28,30].

While the role of PKD3 in cancer has been extensively studied across multiple cancer types, its specific functions in cell immortalization remain largely uncharacterized. Previous work on MEF spontaneous immortalization has implicated PKD3 as a cell cycle regulator after immortalization, where *Prkd3* deficiency was reported to reduce cell proliferation in immortalized MEFs through the modulation of microtubule (MT) dynamics [31]. However, further studies are required to elucidate the mechanisms by which PKD3 is involved in this process. Considering the biological relevance of spontaneous immortalization during carcinogenesis, understanding the mechanisms, pathways and the role of key players such as PKD3 will allow for a better understanding of the multistep process of tumorigenesis. Taking into consideration its role as an upstream regulator of multiple signaling pathways associated with survival and proliferation, our aim is to investigate the temporal changes in the transcriptome landscape regulated by PKD3 in MEFs associated to immortalization. Therefore, we performed a comparative transcriptomic analysis of spontaneously immortalized wild-type (WT) and *Prkd3*-/- MEFs in vitro.

## 2. Results

### 2.1. PKD3 Regulates Ion Homeostasis and Protein Localization in Both Primary and Spontaneously Immortalized MEFs

**Figure 1:**
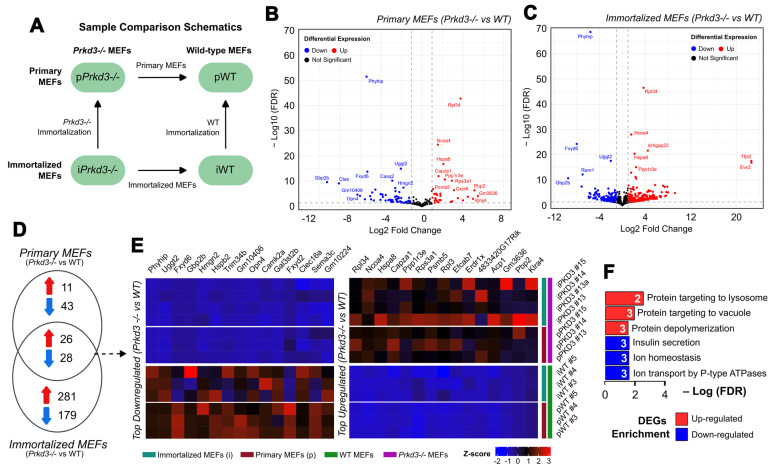
*Prkd3*-regulated transcriptome profile in primary and immortalized MEFs. (**A**) Schematic diagram represents the comparisons between primary and immortalized WT and *Prkd3*-/- MEFs performed in this study. (**B**) Volcano plot of *Prkd3*-regulated DEGs in primary MEFs (*Prkd3*-/- pMEFs vs. WT pMEFs). Volcano plots show the up- and downregulated differentially expressed genes in RNA-seq data with an FDR cut-off < 0.05. Blue dots (left) indicate downregulated DEGs, and red dots (right) indicate upregulated DEGs. (**C**) Volcano plot of *Prkd3*-regulated DEGs in immortalized MEFs (*Prkd3*-/- iMEFs vs. WT iMEFs). (**D**) Venn diagram of 54 overlapping DEGs between *Prkd3*-regulated transcriptome in primary MEFs and spontaneously immortalized MEFs (26 upregulated, 28 downregulated). (**E**) Top 30 most differentially expressed *Prkd3*-regulated genes in MEFs in both primary and spontaneously immortalized states (overlapping for both states). (**F**) Top enriched Gene Ontologies terms and Pathways associated with *Prkd3*-regulated DEGs in both primary and immortalized MEFs, generated by gProfiler.

Spontaneous immortalization is a dynamic process in which cells undergo significant changes in their gene expression [32,33,34,35]. Our recent work predicts phenotypic differences during spontaneous immortalization in MEFs related to cell proliferation, cell adhesion and transcriptional regulation [13]. To understand the role of *Prkd3* in regulating gene expression during spontaneous cell immortalization, we characterized changes in the poly-A transcriptome during this process under WT and *Prkd3*-deficiency conditions using an MEF model. Using a modified 3T3 protocol, we generated three immortalized MEF cell lines from three independent primary WT and *Prkd3*-/- MEF clones (characterized in Figure S1) and collected RNA samples for RNA-seq at two time points: before (primary) and after immortalization. In our study, we performed multiple comparisons between primary and immortalized samples for WT and *Prkd3*-/- genotype MEFs to identify both immortalization status-dependent and immortalization status-independent differentially expressed genes (DEGs); these comparisons are shown in Figure 1A.

We first identified the individual transcriptome profile of *Prkd3*-regulated DEGs for both primary and spontaneously immortalized MEFs. During the primary state (pMEFs), we identified a total of 108 DEGs in *Prkd3*-deficient pMEFs, comprising 71 downregulated genes (shown in blue) and 37 upregulated genes (shown in red) (Figure 1B). After spontaneous immortalization, as shown in the volcano plot (Figure 1C), transcriptome changes in *Prkd3*-deficient immortalized MEFs (iMEFs) compared to their wild-type counterparts account for 514 DEGs, 207 downregulated genes (shown in blue) and 307 upregulated genes (shown in red). It is noteworthy that *Tfpi2* and *Evx2* are the two genes with the highest log2 fold change in *Prkd3*-/- iMEFs. Overall, our transcriptomic analysis suggests that, after spontaneous immortalization in MEFs, *Prkd3* increases the number of potential downstream targets as well as the number of potential immortalization status-dependent biological processes it plays a role in. A complete list of all total DEGs for both primary and immortalized MEF comparisons can be found in Tables S1 and S2, respectively. Further gene ontology analyses for the individual primary and immortalized gene sets are shown in Table S3a and S3b, respectively.

To identify and characterize the genes regulated by *Prkd3* independently of their immortalization state, we perfomed a comparison of the *Prkd3*-regulated transcriptomes in both primary and spontaneously immortalized MEFs, where we show an overlap of 54 DEGs (26 upregulated and 28 downregulated genes) (Figure 1D). The most significantly upregulated DEGs regulated during *Prkd3* deficiency in both primary and immortalized MEFs include *Phyphip*, *Uggt2* and *Fxyd6*, while the most significantly downregulated genes include *Rpl34*, *Ncoa4* and *Hspa8* (Figure 1E). To further elucidate the biological functions associated with the identified DEGs, we performed a GO overrepresentation analysis. These overlapping upregulated DEGs represent genes involved in protein targeting to the lysozome and vacuole, and the downregulated counterparts are associated with ion homeostasis and transport regulated by ATPases (Figure 1F). Our results suggest that, independently of their immortalization status, *Prkd3* may play a role in regulating ion and protein transport and localization in MEFs. The full list of shared genes is provided in Table S4.

### 2.2. After Spontaneous Immortalization in MEFs, PKD3 Regulates the Expression of Genes Associated with Cellular Response to Growth Factor Stimulus, Immune Response and Calcium Ion Homeostasis and Transport

**Figure 2:**
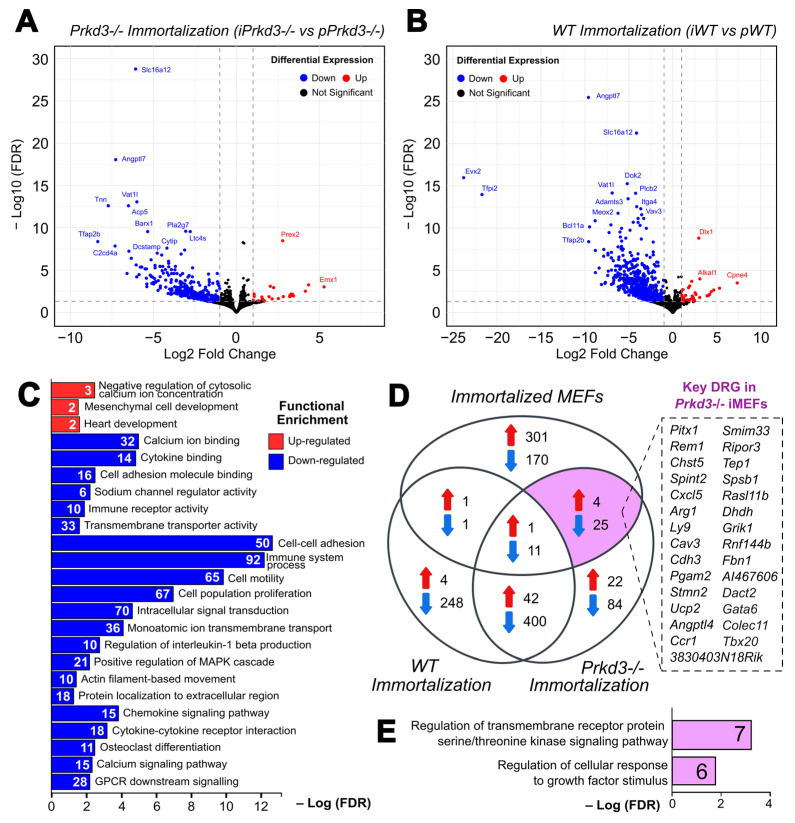
Characterization of transcriptome changes during *Prkd3*-/- MEF immortalization. (**A**) Volcano plot of the immortalization process in *Prkd3*-/- MEFs (*Prkd3*-/- iMEFs vs. *Prkd3*-/- pMEFs). Volcano plots show the up- and downregulated differentially expressed genes in RNA-seq data. Dots in blue (left) indicate downregulated DEGs, and dots in red (right) indicate upregulated DEGs. (**B**) Volcano plot of the immortalization process in WT MEFs (WT iMEFs vs. WT pMEFs). Volcano plots show the up- and downregulated differentially expressed genes in RNA-seq data. Dots in blue (left) indicate downregulated DEGs, and dots in red (right) indicate upregulated DEGs. (**C**) Top enriched Gene Ontologies terms and pathways associated with spontaneous immortalization in *Prkd3*-/- MEFs (*Prkd3*-/- iMEFs vs. *Prkd3*-/- pMEFs), generated by gProfiler. (**D**) Venn diagram of the overlapping DEGs in the gene sets: “Immortalized MEFs” and “Prkd3-/- Immortalization” but not found in “WT Immortalization”. These genes were labeled as key *Prkd3*-regulated genes during spontaneous immortalization (shown in the purple section of the diagram). (**E**) Top enriched Gene Ontologies terms and pathways associated with the key *Prkd3*-regulated genes during spontaneous immortalization, generated by gProfiler.

To address the potential role of *Prkd3* in the temporal regulation of gene expression during spontaneous immortalization, we performed a comparison between iMEFs and pMEFs for both genotypes: WT and *Prkd3*-deficient. In the case of spontaneous immortalization during *Prkd3* deficiency, we identified 369 downregulated genes and 31 upregulated genes, for a total of 400 DEGs (Figure 2A), whereas in WT conditions, we observed a total of 709 DEGs: 661 downregulated and 48 upregulated genes (Figure 2B). The complete lists of all total up- and downregulated genes for both *Prkd3*-/- MEF immortalization and WT MEF immortalization can be found in Tables S5 and S6, respectively.

To assess the biological relevance of the DEGs identified during *Prkd3*-/- MEF immortalization, we performed a gene ontology analysis. The molecular functions, biological processes and pathways enriched among the upregulated genes include negative regulation of cytosolic calcium ion concentration, highlighted by genes such as *Smpd3, Npy1r* and *Bcl2*; and mesenchymal cell development, evidenced by genes like *Bcl2* and *Tbx20* (Figure 2C). In terms of downregulated genes, calcium ion binding (*Plcb2*, *Stab1* and *Hmcn1*), cell adhesion molecule binding (*Tnn*, *Cdh15* and *Cdh3*) and sodium channel regulator activity (*Pcsk9*, *Nos1* and *Scn2b*) represent some of the reduced molecular functions in *Prkd3*-/- iMEFs. Similarly, some of the biological processes associated with the downregulated DEGs include cell–cell adhesion (*Cdh3*, *Pcdhac2* and *Pcdh10*), monoatomic ion transmembrane transport (*Kcnc3*, *Cacna1h* and *Cav3*) and actin filament-based movement (*Gja5*, *Scn1a* and *Scn2b*). Similarly, some of the pathways identified within the downregulated DEGs include osteoclast differentiation (*Acp5*, *Sirpb1a* and *Oscar*), calcium signaling pathway (*Cacna1h*, *P2rx7* and *Plcb2*) and GPCR downstream signaling (*Adcyap1*, *Calcr* and *Vav3*). The list of Gene Ontology terms and their associated genes, generated by gProfiler, is shown in Table S7.

Considering that the reduced proliferative phenotype is observed after immortalization only in *Prkd3*-/- MEFs, we further proceeded to identify key differentially regulated genes (Key DRGs) relevant in *Prkd3*-/- iMEFs. These Key DRGs were recovered from the intersection of two of our previous gene sets: (1) the DEGs from the comparison “*Prkd3*-/- Immortalization” (refer to Figure 2A) and (2) the DEGs from the comparison “Immortalized MEFs” (refer to Figure 1C); at the same time, these excluded the DEGs that intersected those in the comparison “WT Immortalization”. These genes represent potential downstream targets regulated by PKD3 during this process. To this end, we performed a Venn diagram with the three datasets, where we recovered 29 key DRGs in *Prkd3*-/- iMEFs: 25 downregulated and 4 upregulated (section highlighted in purple, Figure 2D). These key DRGs play a role in the transmembrane receptor protein serine/threonine kinase signaling pathway, such as *Cav3*, *Raslb11b* and *Fbn1*, and regulation of cellular response to growth factor stimulus, including *Dact2*, *Gata6* and *Tbx20* (Figure 2E).

**Figure 3:**
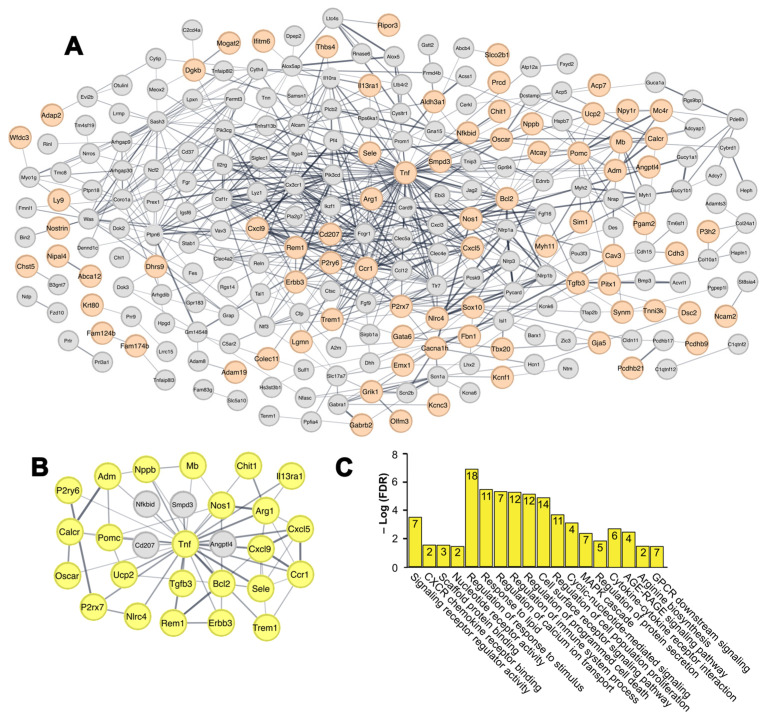
Identification and characterization of hub genes associated with *Prkd3* deficiency in spontaneously immortalized MEFs. (**A**) Protein–protein interaction (PPI) network of the full list of DEGs identified during spontaneous immortalization in *Prkd3*-/- MEFs, generated using the STRING database. Orange nodes indicate DEGs identified only in *Prkd3*-/- MEF spontaneous immortalization and not during its WT counterpart. (**B**) The highest score clustering module of hub genes generated by MCODE from the full DEG PPI network, yellow nodes indicate the gene hubs associated to spontaneous immortalization in *Prkd3*-/- MEFs, identified in the overlap of both MCODE and Cytohubba algorithms. (**C**) Gene Ontology and Kegg Pathway terms related to the newly identified FPDSP hub genes associated with *Prkd3* deficiency in spontaneously immortalized MEFs, generated by gProfiler.

We further performed a protein–protein interaction analysis using the STRING database to explore the relationship between the DEGs identified during spontaneous immortalization in *Prkd3*-deficient MEFs. These DEGs were constructed into a network consisting of 392 gene nodes and 635 interaction edges, where 79 proteins were highlighted in orange because they were identified only during spontaneous immortalization in *Prkd3*-/- MEFs and not in WT immortalization (Figure 3A). This network allows for us to observe the interplay between the genes involved in the intrinsic process of spontaneous immortalization and those regulated by *Prkd3* during this process.

Subsequently, we identified potential hub genes within the genes observed only during *Prkd3*-/- immortalization. These hub genes are considered important players in the regulation of the network and may play a significant role in multiple biological pathways. In our case, these genes represent relevant targets during the process of spontaneous immortalization in *Prkd3*-deficient MEFs. To do so, we used the molecular complex detection (MCODE) algorithm, a theoretical cluster algorithm that allows for the identification of densely connected regions, and further verified the identified hub genes using the topological analysis methods of the Cytohubba hub identifying algorithm, which allowed for us to identify the genes with the highest interconnectivity within the PPI network [36,37]. Using the MCODE algorithm, we obtained a hub gene cluster network composed of 28 gene nodes (MCODE score: 4.44) (Figure 3B), where 24 hub genes are in the intersection of the MCODE and Cytohubba algorithms (shown as yellow nodes in Figure 3B). These hub genes regulated by *Prkd3* during spontaneous immortalization in MEF correspond to adrenomedullin (*Adm*), arginase (*Arg1*), identified B-cell leukemia/lymphoma 2 (*Bcl2*), calcitonin receptor (*Calcr*), C-C motif chemokine receptor 1 (*Ccr1*), chitinase 1 (*Chit1*), C-X-C motif chemokine ligand 5 (*Cxcl5*), C-X-C motif chemokine ligand 9 (*Cxcl9*), erb-b2 receptor tyrosine kinase 3 (*Erbb3*), interleukin 13 receptor alpha 1 (*Il13ra1*), myoglobin (*Mb*), NLR family CARD domain containing 4 (*Nlrc4*), nitric oxide synthase 1 (*Nos1*), natriuretic peptide type B (*Nppb*), osteoclast associated receptor (*Oscar*), purinergic receptor P2X, ligand-gated ion channel 7 (*P2rx7*), pyrimidinergic receptor P2Y 6 (*P2ry6*), pro-opiomelanocortin-alpha (*Pomc*), rad and gem related GTP binding protein 1 (*Rem1*), selectin (*Sele*), transforming growth factor beta 3 (*Tgfb3*), triggering receptor expressed on myeloid cells 1 (*Trem1*), tumor necrosis factor (*Tnf*) and uncoupling protein 2 (*Ucp2*).

Some of the relevant molecular functions associated to these hub genes include CXCR chemokine receptor binding (*Cxcl9* and *Cxcl5*), scaffold protein binding (*Nos1*, *P2rx7* and *Trem1*) and nucleotide receptor activity (*P2rx7* and *P2ry6*) (Figure 3C). In terms of biological processes, regulation of calcium ion transport (*P2rx7*, *Rem1* and *Calcr*), regulation of immune system processes (*Tnf*, *Ccr1* and *Pomc*), regulation of programmed cell death (*Adm*, *Nos1* and *Tnf*) and regulation of cell population proliferation (*Tnf*, *Tgfb3* and *Erbb3*) are major processes enriched within these hub genes. Finally, some of the pathways associated with the hub genes include the AGE-RAGE signaling pathway (*Tnf*, *Sele* and *Tgfb3*), arginine biosynthesis (*Nos1* and *Arg1*) and GPCR downstream signaling (*Pomc*, *P2ry6* and *Calcr*). The list of the Gene Ontology terms and their associated genes, generated by gProfiler, are shown in Table S8**.**

### 2.3. Predicted Gene Regulatory Network Shows a High Level of Interaction Between PKD3-Regulated Genes Post-Spontaneous Immortalization in MEFs

**Figure 4:**
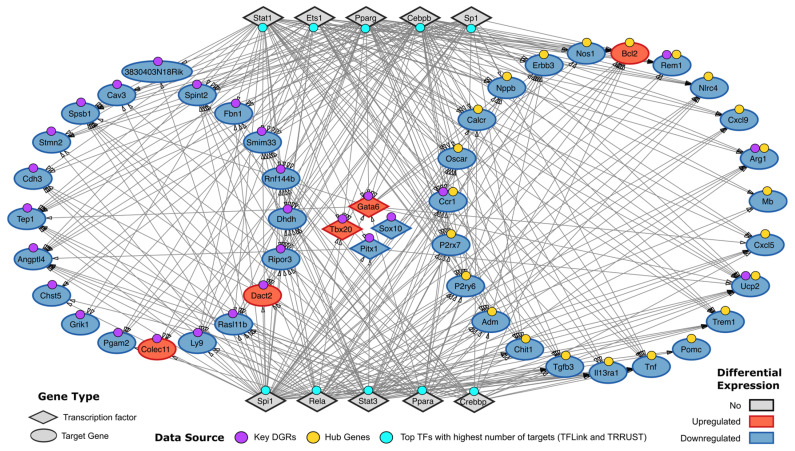
Predicted TF-target gene regulatory network associated with spontaneous immortalization in *Prkd3*-deficient MEFs. Gene types are shown in different shapes: Diamond (Transcription factor) and Ellipse (Target gene). Differential expression of our genes of interest are shown in different colors: Gray (No Differential expression), Red (Upregulated genes) and Blue (Downregulated genes). Our gene sets of interest or data sources are shown with different color tags: Purple tag (Key DRG list), Yellow (Hub genes) and Cyan (Top TFs with the highest number of targets found in both TFLink and TRRUST databases). Arrows indicate the direction of the regulation—from TF to target gene.

To investigate the potential regulatory systems within the relevant genes associated with PKD3 after spontaneous immortalization, we integrated the key DRGs and hub genes identified in our previous analysis into a PKD3-regulated post-immortalization gene regulatory network. This network allowed for us to visualize the potential interactions between transcription factors (TFs) and target genes differentially regulated by PKD3 during the process of immortalization. To do this, we extracted the predicted TF-target gene interactions of our genes using the TFLink and TRRUST databases, repositories with small- and large-scale experimental evidence of regulatory interactions. Within our network, we identified the top five target genes with the highest number of predicted TFs: *Ucp2*, *Angptl4*, *Smim33*, *Spsb1* and *Spint2* (Key DRGs), and *Ucp2*, *Blc2*, *Tnf*, *Adm* and *Chit1* (Hub Genes) (Figure 4). In addition, we retrieved the top five TFs with the highest number of predicted targets, corresponding to *Spi1*, *Pparg*, *Cebpb*, *Stat1* and *Stat3*.

## 3. Discussion

Cell immortalization is a clinically significant phenotype observed in several cancers, characterized by the acquisition of unlimited proliferative potential—a hallmark of cancer progression. Oncogenic alterations that contribute to this phenotype have been extensively studied in various models, including mouse embryonic fibroblasts (MEFs). The reactivation of cellular mechanisms that facilitate cell cycle re-entry is typically associated with the accumulation of genetic alterations and changes in gene expression profiles. Numerous studies attempted to elucidate the underlying mechanisms of MEF immortalization; previous analyses suggest that spontaneous immortalization is accompanied by changes in proliferation, cell adhesion and transcriptional programs, with particular relevance to MAPK signaling pathways and cadherin molecules [13,38]; however, the specific involvement of *Prkd3* in this process remains unclear.

PKD3 is considered an oncogene that is frequently dysregulated in human cancers and regulates multiple oncogenic processes. While its role in immortalization is poorly described, Zhang et al. (2013) identified Prkd3 as a critical regulator of proliferation rates following spontaneous immortalization in MEFs [31]. Consistent with these observations, several studies using different cancer cell lines have proposed that PKD3 has distinct functions in cell cycle regulation. Chen et al. (2008) demonstrated that PKD3 modulates the Akt and Erk1/2 signaling pathways in PC cells, contributing to enhanced cell growth [28]. Furthermore, Hao et al. (2013) showed that PKD3 is highly upregulated in triple-negative BC, and its knockdown resulted in a significant reduction in cell proliferation, exerting inhibitory effects on cell growth [39]. Since cell cycle control and proliferation are key features of this process, our study aimed to identify potential processes and genes regulated by *Prkd3* that may contribute to proliferation modulation post-spontaneous immortalization.

We hypothesize that the cell cycle and proliferation modulation in primary MEF cells are regulated by multiple redundant pathways. However, this system undergoes a shift during immortalization, resulting in a *Prkd3* dependent pathway. Our transcriptomic analysis suggests that *Prkd3* may regulate several key processes involved in cell proliferation following spontaneous immortalization. Within our analysis, we highlight a number of differentially regulated genes in immortalized *Prkd3*-deficient MEFs compared to both primary *Prkd3*-deficient MEFs and immortalized wild-type MEFs as well as a cluster of novel hub genes regulated by *Prkd3* during this process. Collectively, our study suggests that *Prkd3* is involved in the downregulation of pro-proliferative glycolitic enzymes, calcium homeostasis mediators and microtubule dynamics regulators at the intersection of the PI3K/Akt and MAPK pathways in MEFs following spontaneous immortalization.

We have previoulsy proposed the p38 MAPK and PI3K/AKT signaling pathways as important regulators of proliferation following spontaneous immortalization in MEFs [13]. Previous studies have shown that these pathways interact with the PKC/PKD signaling pathway, with both p38 and AKT kinases acting as upstream regulators of PKD3 [28,40]. We propose three key DRGs, mediators of the MAPK and AKT pathways, as glycolysis-associated pro-proliferative genes that are downregulated upon immortalization due to *Prkd3* deficiency: phosphoglycerate mutase (*Pgam2*), *Ucp2* and *Angptl4*. (1) Mikawa et al. (2020) showed that *Pgam2* is essential to induce Warburg glycolytic proliferation in cancer cells, supported by Kondoh et al. (2005) who showed that the overexpression of *Pgam2* enhances glycolysis in MEFs [41,42]. *PGAM2* is highly expressed in HCC and PC, and its inhibition has been shown to reduce cell proliferation and tumor growth in HCC and mouse xenograft tumor models [43,44,45]. (2) Research has shown that *UCP2* can drive a metabolic shift towards increased glycolysis in non-small-cell lung cancer (NSCLC), and its overexpression also enhances glycolysis during skin transformation [46,47]. Clinically, upregulation of *UCP2* has been observed in NSCLC tissues compared to adjacent normal tissues as well as in glioma (GLC) and gallbladder cancer [46,48,49]. UCP2 has been linked to the p38 MAPK pathway, where its upregulation increases proliferation and invasion, whereas its inhibition reduces chemoresistance and promotes apoptosis [48]. (3) Elevated levels of ANGPTL4 have been shown to promote glucose uptake and stimulate glycolysis in both endothelial cells and CRC cells in vitro and in vivo [50,51]. Overexpression of ANGPTL4 promotes proliferation and migration and inhibits cell cycle arrest in thyroid, ovarian and squamous cell carcinoma and osteosarcoma, while suppression of ANGPTL4 reverses this behavior [52,53,54]. Interestingly, Loza-Valdez showed that PKD3 controls glucose metabolism and promotes insulin sensitivity [55]. In addition, PKD3 has been shown to promote GC proliferation associated with enhanced glycolysis [25].

Furthermore, our analysis reveals TNF as the most connected gene hub, which is only downregulated during immortalization under *Prkd3* deficiency, highlighting its potential relevance in mediating several of the biological processes regulated by *Prkd3*. TNF is a key mediator of the NFκB and MAPK signaling pathways involved in proliferation, cell death and survival. This cytokine has been a target of study for several decades due to its dual role in cancer for both inducing and inhibiting tumor progression, which has been described as cellular context specific. In terms of its regulation, Kentny et al. (1998) demonstrated that PKCs, including the upstream activator of PKD3 PKCε, activate MAPK to induce the expression of TNF [56,57]. Several studies have shown that PKD3 activates both STAT3 and NFκB, which are known to upregulate the expression and induce the pro-proliferative and anti-apoptotic activity of TNF signaling [40,57,58,59]. Interestingly, STAT3 corrresponds to one of the top five TF to be involved in predicted GRN associated to immortalization in Prkd3-deficient MEFs. As reviewed by Grivennikov and Karin (2010), NF-κB and STAT3 synergistically contribute to the initiation and progression of CRC, GC and LC [60]. Therefore, we propose that PKD3 regulates TNF expression in an oncogenic manner, whereas *Prkd3* deficiency results in reduced proliferation, which may be influenced by *Tnf* depletion. Furthermore, the link between TNF and glycolysis has been previously reported in several cell types. In mesenchymal stem cells, TNF has been shown to activate PI3K-Akt signaling to drive glycolysis, and in prostate epithelial cells, TNF was demonstrated to increase aerobic glycolysis and reduce oxidative metabolism [61,62]. TNF has also been shown to induce glycolytic shift and promote proliferation [63]. Additionally, TNF has been shown to positively regulate UCP2 and PGAM2 expression [43,64,65], and ANGPTL4 expression has been hypothesized to be regulated by and highly correlated with TNF [66]. Therefore, we propose that *Tnf* acts as an intermediate mediator of these glycolytic molecules, induced by *Prkd3*.

Secondly, an important process highlighted within our study and potentially regulated by *Prkd3* after spontaneous immortalization corresponds to calcium homeostasis and transport. Throughout our analysis, we observe calcium-related ontologies associated with DEGs regulated by *Prkd3* after spontaneous immortalization as well as highlighted in our hub genes. Transient increases in cytosolic calcium concentrations are critical for cell division and proliferation, dynamics controlled by cellular influx and release from endoplasmic reticulum (ER) Ca^2+^ stores. Our results suggest that *Prkd3* modulates calcium homeostasis by regulating the expression of *Calcr* and *P2ry7*. (1) CALCR has been shown to couple to several heterotrimeric G-proteins, which initiate the phospholipase C (PLC) pathway and induce the release of Ca^2+^ from intracellular stores and promote an influx of extracellular calcium [67,68]. This increase in intracellular calcium partially regulates MAPK signaling in a PKC-dependent manner [69]. CALCR expression has been identified in several cancer cell lines, including those derived from BC, PC, bone cancer, multiple myeloma, leukemia (LKM) and glioblastoma (GBM) [70]. In vitro studies have shown that it induces oncogenic properties, such as increased proliferation and migration in NSCLC, PC and renal cancer (RC), while its knockdown induces apoptosis and cell cycle arrest. Clinical data also show its correlation with cancer progression and unfavorable prognosis in RC [71,72,73]. (2) P2rx7 is an ATP-gated receptor responsible for calcium influx in a variety of cell types. P2rx7-mediated calcium signaling has been shown to trigger the activation of intracellular pathways, including MAPK and PI3K/AKT, critical for cancer proliferation and survival (reviewed in [74,75]). According to Young et al. (2017), up-expression of this receptor was observed in all tumor types reported in The Cancer Genome Atlas [76]. Both in vitro and in vivo studies have shown that increased P2rx7 activity induces proliferation in GLC, LKM and pancreatic cancer (PCC), and its blockade reduces tumor burden in several cancer types and experimental models [77,78,79,80,81,82]. Dysregulated Ca^2+^ signaling is implicated in malignant transformation. A reduction in endoplasmic reticulum Ca^2+^ levels often confers resistance to apoptosis. This is accompanied by increased Ca^2+^ entry, which increases cytosolic Ca^2+^ levels and induces oncogenic changes in gene expression and cell cycle regulation [83,84,85]. Accordingly, using the HEK293 model, PKD3 has been shown to modulate the oscillation of intracellular calcium in conjunction with PKD1 [86]. It has also been reported that activated PKCs enhance calcium influx in neurons [87]. Therefore, we propose that PKD3 may play a potential role in calcium homeostasis by regulating the expression of *Calcr* and *P2rx7* to regulate MEF proliferation after immortalization.

Using our immortalized *Prkd3*-/- MEF model, Zhang et al. (2016) showed that PKD3 regulates both MT nucleation and polymerization/depolymerization dynamics [31]. Furthermore, Papazyan et al. (2008) reported that PKD3 and PKD2 localize to MT structures during mitosis [21]. These studies highlight the importance of PKD3 in regulating MT dynamics during proliferation. In our study, we identified Stathmin 2 (*Stmn2*) and microtubule associated protein 10 (*Map10*), which encode MT-interacting proteins and are downregulated after immortalization in *Prkd3*-deficient MEFs. (1) STMN2 is an important regulator of MT disassembly that has been shown to play a role in cancer development. High expression of *STMN2* has been reported to be associated with PCC and HCC progression as well as hypertrophic scar fibrosis [88,89,90]. These reports showed that overexpression of *STMN2* induced cell proliferation and migration, a behavior that was reversed by its inhibition. In addition, *STMN2* overexpression predicts poor survival in NSCLC and OV [91,92]. (2) *Map10* is a novel microtubule stability modulator with high relevance for cytokinesis completion [93]. While functional studies on Map10 and its relationship to cancer development are limited, Shakya et al. (2024) recently suggested a link between cytoskeletal and metabolic alterations that promote proliferation and migration in metastatic PC with *Map10* upregulation [94]. Given that several reports suggest that cancer progression is associated with aberrant MT dynamics and the expression of MT dynamics regulators expression, we propose that *Prkd3* regulates the expression of the MT-associated proteins *Stmn2* and *Map10*, favoring a proliferative phenotype in immortalized MEFs.

Moreover, an intriguing potential target candidate regulated by PKD3 in our study is tissue factor pathway inhibitor 2 (*Tfpi2*), a protease inhibitor. *Tfpi2* is recognized as an important tumor suppressor associated with extracellular matrix (ECM) modulation and plays a critical role in tumor invasion and metastasis as well as proliferation, adhesion and apoptosis [95,96,97]. Our results show that *Prkd3*-deficient immortalized mouse embryonic fibroblasts (MEFs) exhibit higher levels of TFPI-2 expression compared to their wild-type counterparts. Notably, spontaneous immortalization in the presence of *Prkd3* results in a significant reduction in *Tfpi2* expression. Consistent with these observations, reduced *Tfpi2* expression has been correlated with cancer progression, recurrence and poor prognosis, and it is downregulated in several aggressive tumors, including BC, CRC, PC, HCC, PCC and NSCLC [98,99,100,101,102,103]. Evidence suggests that *Tfpi2* may exert pro-apoptotic effects in low-grade GLC and GBM, with its expression inversely correlated with the proliferative index (Ki-67) [104,105]. *Tfpi2* has been shown to interact with the transcription factor AP-2, thereby inhibiting BC cell proliferation and invasion [106], with similar proliferative inhibitory effects in HCC [101]. Additionally, *Tfpi2* reduces ECM proteolysis; thus, its upregulation counteracts ECM degradation by cancer cells and inhibits cell invasion [97,99]. Regarding its regulation, epigenetic silencing has been frequently described in metastatic cancers compared to localized tumors [107,108], and suppression of the ERK and AKT pathways contributes to the *Tfpi2*-mediated proliferation inhibition [109].

**Figure 5:**
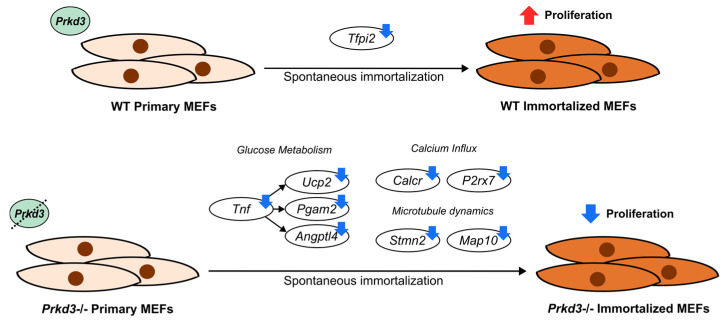
Transcriptomic analysis suggests a potential model for altered biological processes (glucose metabolism, calcium influx and microtubule dynamics) and genes regulated by *Prkd3* involved in the modulation of MEF proliferation after spontaneous immortalization.

Taken together, our study proposes a potential model for the *Prkd3*-induced regulation of MEFs after spontaneous immortalization, characterized by the downregulation of genes involved in three different biological processes: glucose metabolism (*Tnf*, *Ucp2*, *Pgam3* and *Angptl4*), calcium homeostasis (*Calcr* and *P2rx7*) and microtubule dynamics (*Stmn2* and *Map10*) (Figure 5). Several studies have extensively described the role of these processes in the regulation of proliferation and cancer development. In addition, we suggest the involvement of the tumor suppressor gene *Tfpi2* in modulating proliferation as a downstream target of *Prkd3*. We note that further functional and validation assays as well as in vivo studies in both MEFs and human models are required to confirm the role of the proposed genes and processes in regulating proliferation after spontaneous immortalization and their involvement in carcinogenesis. Investigation of the transcriptomic changes regulated by *Prkd3* in spontaneously immortalized cells is crucial to identify biological markers involved in proliferation that could predict the early risk of cellular transformation associated with cancer development.

## 4. Materials and Methods

### 4.1. Cell Culture and Immortalization

*Prkd3*-deficient mice mutants were generated by Zhang et al. (2016) [31]. Primary mouse embryonic fibroblasts (pMEFs) were previously recovered from wild-type and the previously validated *Prkd3*-deficient mice by our lab from 3 different embryos in Zhang et al.’s (2016) study [31] using standard protocols [8]. Cells were cultured in Dulbecco’s Modified Eagle’s Medium (DMEM)—high glucose (Sigma-Aldrich, Oakville, ON, Canada, Cat#D5796-500ML) supplemented with 10% fetal calf serum (FCS) (FisherScientific, Nepean, ON, Canada, Cytiva Cat#SH3412IH345), 1X non-essential amino acids (FisherScientific, Mississauga, ON, Canada, Cytiva Cat#SH30238.01), 1% Pen Strep (FisherScientific, Mississauga, ON, Canada, Gibco Cat#15070-063) and β-mercaptoethanol (FisherScientific, Mississauga, ON, Canada, Gibco Cat# 21985023).

Primary cell culture and the modified NIH 3T3 spontaneous immortalization protocols were followed as described by Loaiza-Moss et al. [13]. Cryopreserved pMEFs (passage 0) were thawed and plated onto 10 cm culture dishes and maintained under standard conditions at 37 °C with 5% CO_2_. Cultures were serially passaged at approximately 80% confluence using a 1:3 split ratio until the cells entered senescence, which typically occurred after 5–6 passages. Upon reaching senescence, the culture medium was switched to an MEF culture medium supplemented with 20% FCS, with media changes performed every 3 days. Prolonged culturing under these conditions led to the emergence of spontaneously immortalized cells, observed as distinct growing colonies. These colonies were subsequently expanded, establishing immortalized MEF cell lines (iMEFs). Following immortalization, the iMEFs were cultured and propagated in MEF culture medium containing 10% FCS to ensure optimal growth and maintenance.

### 4.2. RNA Sequencing and Differential Expression Analysis

RNA sequencing was performed on three biological pMEF and iMEF samples. MEF total RNA was extracted using the AllPrep DNA/RNA Mini Kit (Qiagen, Toronto, ON, Canada, Cat#80204). Poly-A-enriched RNA library preparation and sequencing were performed by the Centre d’expertise et de services Génome Québec (Montreal, QC, Canada) on the Illumina NovaSeq 6000 platform. FASTQ file quality was assessed using FASTQC software (version 0.12.0). Pair-end reads were aligned to the mm10 (mouse) reference genome employing the STAR software (version 2.7.10a) [110]. Subsequently, filtering and indexing were performed with Samtools (version 1.18) [111]. After processing, the gene counts were assigned using the featureCounts function of Subread (version 2.0.6) [112] based on annotated genes in the Ensembl GRCm38 assembly. Differentially expressed genes (DEGs) were identified using the R software Bioconductor DESeq2 package (version 1.41.13) [113] using a standard threshold of FDR-adjusted *p* values < 0.05 and L2FC of ±1. The data files for RNA-seq have been deposited in the NCBI’s Gene Expression Omnibus [114] and are accessible through GEO Series accession number: GSE266445.

### 4.3. Gene Ontology and Pathway Enrichment Analysis

Gene Ontology (GO) analysis [115,116] for the categories of biological process (BP) and molecular function (MF) and pathway over-representation analysis based on Kyoto Encyclopedia of Genes and Genomes (KEGG) database [117,118] were carried out for differentially expressed genes using the online database platform gProfiler [119], with a corrected *p* value < 0.05 cut-off.

### 4.4. Protein–Protein Interaction (PPI) Analysis and Hub Gene Identification

A PPI network of DEGs was constructed using the STRING database (version 2.0.2) and was visualized in Cytoscape (version 3.10.1) [120,121]. Gene hubs were identified using a modified pipeline of that shown by Ma et al. [122]. Gene hubs were selected based on the highest scoring module calculated using the MCODE hub analysis [37] and further verified using the 11 topological analysis methods employing the Cytohubba Cytoscape plug-in (MCC, DMNC, MNC, Degree, EPC, Bottleneck, EcCentricity, Closeness, Radiality, Betweenness and Stress) [36].

### 4.5. Transcription Factor (TF)—Target Gene Regulatory Network

Transcription factors were identified using Gifford-lab / Reprogramming Recovery Github repository [123] and the mouse AnimalTFDB 3.0 database [124], as described by Henze et al. [125]. TF-target gene interactions were obtained from the TFLink and TRRUST (version 2) databases [126,127] and visualized using Cytoscape.

## Data Availability

RNA-seq data presented in this study are openly available in GEO (Series GSE266445).

## Supplementary Materials

The following supporting information can be downloaded at: https://www.mdpi.com/article/10.3390/ijms26020596/s1.
